# [^18^F]FE@SNAP—a specific PET tracer for melanin-concentrating hormone receptor 1 imaging?

**DOI:** 10.1186/s13550-016-0186-7

**Published:** 2016-04-01

**Authors:** Cécile Philippe, Daniela Haeusler, Thomas Scherer, Clemens Fürnsinn, Markus Zeilinger, Wolfgang Wadsak, Karem Shanab, Helmut Spreitzer, Marcus Hacker, Markus Mitterhauser

**Affiliations:** Department of Biomedical Imaging and Image-guided Therapy, Division of Nuclear Medicine, Medical University of Vienna, Waehringer Guertel 18-20, 1090 Vienna, Austria; Department of Pharmaceutical Technology and Biopharmaceutics, University of Vienna, Vienna, Austria; Department of Medicine III, Division of Endocrinology and Metabolism, Medical University of Vienna, Vienna, Austria; Department of Pharmaceutical Chemistry, University of Vienna, Vienna, Austria; Ludwig Boltzmann Institute for Applied Diagnostics, Vienna, Austria

**Keywords:** [^18^F]FE@SNAP, MCHR1, Autoradiography, In vivo, Imaging

## Abstract

**Background:**

The melanin-concentrating hormone receptor 1 (MCHR1), which is highly expressed in the lateral hypothalamus, plays a key role in energy homeostasis, obesity and other endocrine diseases. Hence, there is a major interest in in vivo imaging of this receptor. A PET tracer would allow non-invasive in vivo visualization and quantification of the MCHR1. The aim of the study was the ex vivo evaluation of the MCHR1 ligand [^18^F]FE@SNAP as a potential PET tracer for the MCHR1.

**Methods:**

[^18^F]FE@SNAP was injected directly into the jugular vein of awake naïve rats for ex vivo brain autoradiography, biodistribution and additional blood metabolite analysis. Blocking experiments were conducted using the unlabeled MCHR1 ligand SNAP-7941.

**Results:**

A high uptake of [^18^F]FE@SNAP was observed in the lateral hypothalamus and the ventricular system. Both regions were significantly blocked by SNAP-7941. Biodistribution evinced the highest uptake in the kidneys, adrenals, lung and duodenum. Specific blocking with SNAP-7941 led to a significant tracer reduction in the heart and adrenals. In plasma samples, 47.73 ± 6.1 % of a hydrophilic radioactive metabolite was found 45 min after tracer injection.

**Conclusions:**

Since [^18^F]FE@SNAP uptake was significantly blocked in the lateral hypothalamus, there is strong evidence that [^18^F]FE@SNAP is a highly suitable agent for specific MCHR1 imaging in the central nervous system. Additionally, this finding is supported by the specific blocking in the ventricular system, where the MCHR1 is expressed in the ependymal cells. These findings suggest that [^18^F]FE@SNAP could serve as a useful imaging and therapy monitoring tool for MCHR1-related pathologies.

## Background

The melanin-concentrating hormone receptor 1 (MCHR1) plays a key role in energy homeostasis and obesity [1, 2]. Furthermore, it has been implicated to be involved in the pathogenesis of diabetes [3, 4] and inflammatory processes in the gut [5]. Since obesity affects over 600 million individuals worldwide (as estimated by the World Health Organization in 2014 [6]), there is extensive pharmaceutical interest in the development of anti-obesity drugs. It has been shown that MCHR1 antagonists reduce body weight in rodents [7]. Nevertheless, none of these molecules reached market authorization so far. A MCHR1-positron emission tomography (PET) ligand could support dose selection of MCHR1 antagonists [7] and, therefore, would be a valuable tool for drug development. PET allows non-invasive in vivo visualization and quantification of receptor systems, as well as monitoring and following hormone receptor status and related pathologies in vivo. Besides the application of a MCHR1-PET tracer for compound dose selection of potential MCHR1-targeting drugs, another potential implication for obesity patients could be the in vivo quantification of the MCHR1—which is predominantly expressed in the lateral hypothalamus [8]—as a risk factor and early diagnostic tool for insulin resistance. Furthermore, a MCHR1-PET ligand could help to better understand the endocrine status and guide pharmacological intervention via the MCHR1.

**Fig. 1:**
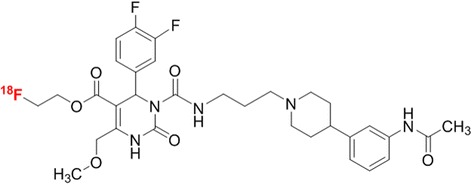
Chemical structure of [^18^F]FE@SNAP. *Red colour* indicates the radiolabel position

So far, based on the specific MCHR1 antagonist SNAP-7941 [9], [^11^C]SNAP-7941 was developed as the first PET tracer for the MCHR1 and was evaluated in a preclinical study [10, 11]. Furthermore, we introduced the ^18^F-fluoroethylated analogue [^18^F]FE@SNAP (Fig. 1) as an alternative potential PET tracer for the MCHR1 [12, 13]. [^18^F]FE@SNAP showed a high affinity (*K*_d_ = 2.9 nM, evaluated on CHO cells expressing the human MCHR1) and selectivity (*K*_i_ > 1000 nM on the second MCH receptor, MCHR2) towards the MCHR1 [13].

After successful in vitro evaluation, the next logical step in the preclinical evaluation process was the performance of ex vivo experiments. Hence, the purpose of the present study was to confirm the potential of [^18^F]FE@SNAP for specific MCHR1 brain imaging in healthy rats. Therefore, [^18^F]FE@SNAP was administered IV for ex vivo brain autoradiography and additionally to study biodistribution and to search for potential circulating metabolites. It is noteworthy that IV application was performed through the jugular vein, allowing animals to be awake and conscious, hence excluding the well-known significant anaesthetic influence on imaging results [14–16].

## Methods

### Animals

With free access to tap water and standard laboratory animal diet, 16-week-old male Sprague-Dawley rats (436 ± 79 g, mean ± SD) were kept under controlled environmental conditions on a 12-h light–12-h dark cycle (Alleinfutter für Ratten und Maeuse sniff R/M-H, sniff Spezialdiaeten GmbH; Soest, Germany).

For implantation with indwelling catheters into the right jugular, rats were anaesthetized by an intraperitoneal injection of ketamine-xylazine supplemented, if necessary, with inhalative sevoflurane [17]. Tracer experiments were performed not earlier than 7 days after surgery, when all rats were ±10 % within their pre-surgical body weight. Since these catheters allowed IV injections into conscious freely moving rats, any influence of anaesthesia was excluded in these experiments. All procedures and protocols using animals have been approved by the Institutional Animal Care and Use Committee of the Medical University of Vienna, Austria, as well as by the Austrian Ministry of Science, Research and Economy (BMWF-66.009/0268-II/3b/2012).

### Tracer preparation

Radiosyntheses of [^18^F]FE@SNAP and [^18^F]altanserin were performed in a microfluidic device (Advion NanoTek®) followed by a purification in a conventional synthesizer (Nuclear Interface®) as described elsewhere [13]. [^18^F]FE@SNAP was formulated in physiological saline solution (606 ± 332 MBq/500 ± 346 μL; formulation volume depending on radiochemical yield).

### IV study including biodistribution and metabolite analysis

Conscious and freely moving rats of the baseline and blocking groups were always examined simultaneously: rats of the baseline group (*n* = 3) received vehicle (400 μL), and rats of the blocking group (*n* = 3) received SNAP-7941 (15 mg/kg; freshly dissolved in 400 μL) via the jugular vein 30 min prior to tracer application. Via the jugular vein, 51.33 ± 26.2 MBq of [^18^F]FE@SNAP (specific activity 12.3–43.1 GBq/μmol; radiochemical purity ≥95 %; 30–100 μL) was then administered to all rats. After 45 min, rats were sacrificed by IV ketamine injection and decapitated, and the brains were removed and immediately quick frozen in isopentane (−45 °C) for ex vivo autoradiography. Other organs including the eyes, tongue, muscle, epidermal white adipose tissue (WATep), heart, lung, stomach, pancreas, liver, duodenum, colon, spleen, kidneys, adrenals, testis, bladder and bone as well as blood and urine were removed, weighed and measured in a gamma counter (2480 WIZARD^2^, PerkinElmer). Radioactivity concentrations were normalized to dose and weight and expressed as percent injected dose per gram (%ID/g). To determine significant differences, a two-tailed *t* test with *α* = 0.95 was performed using the statistics add-on in Microsoft Excel® 2013. A value of *P* < 0.05 was considered as significant.

For analysis of potential circulating metabolites, blood samples from the baseline group were collected into heparinized tubes and immediately stored on ice before processing. The blood was centrifuged (Hettich Rotanta/TRC; 3400×*g*, 4 min) to separate cellular components. Sample cleanup was performed by vortexing plasma with the equivalent amount of acetonitrile and by subsequent centrifugation (Hettich Universal 30RF; 23,000×*g*, 3 min) to remove precipitated proteins. The obtained supernatant was applied to radio-thin-layer chromatography (radio-TLC silica gel plates, mobile phase acetonitrile/water 70/30 *v*/*v*, application volume 2 μL on origin) and analysed via a Canberra-Packard Instant Imager.

### Ex vivo autoradiography

**Fig. 2:**
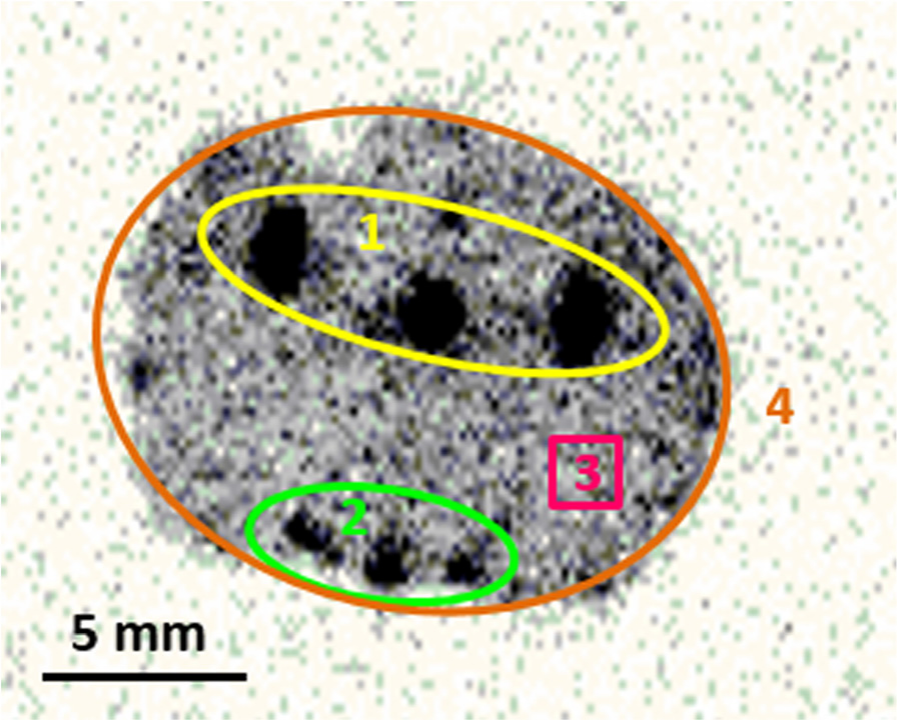
Scheme of the ROIs for calculation of ratios. *1* (*yellow*): ventricular system; *2* (*green*): hypothalamic area; *3* (*pink*): non-target region; and *4* (*orange*): whole brain. It is noteworthy that ROIs 1 and 2 include the target areas as well as some background areas, due to varying target size throughout the different brain levels (bregma)

Whole rat brains (*n* = 6) were cut in a cryo-microtome (Microm HM 560, Thermo Scientific) into 50-μm-thick slices and thaw-mounted onto superfrost slides (Menzel-Gläser SUPERFROST® PLUS, Thermo Scientific). Samples were placed on Phosphor Imager plates (Multisensitive Phosphor Screens Long Type MS, PPN 7001724, PerkinElmer) for an exposure period of 18 h and then analysed with a Cyclone Phosphor Imager (Cyclone Plus Storage Phosphor System, PerkinElmer). Data analysis was performed with OptiQuant® data processing software Version 5.0. Due to high-MCHR1 appearance, special emphasis was laid on the hypothalamus [8] and the ventricular system [18, 19]. Hence, regions of interest (ROIs) were drawn for the hypothalamic region and the ventricle, and additionally, a non-target region and the whole tissue were selected (Fig. 2). To facilitate comparison, manually defined template ROIs fitting to all slices (30 slices/brain; bregma −1.0 to −2.5 mm) were applied. ROIs resulted in normalized digital light units per square millimeter (DLU/mm^2^); values were expressed as ratio of MCHR1-rich and reference region. In detail, ratios of hypothalamus/non-target and ventricle/non-target were calculated. Significant differences were calculated as described before.

### Preliminary small-animal imaging

An anaesthetized rat (with 1.5–2.5 % isoflurane) was immobilized in a multimodal animal carrier unit (MACU; medres®—medical research GmbH, Cologne, Germany) and maintained at a body temperature of 37 °C throughout the whole experiment. [^18^F]FE@SNAP (47.64 ± 1.23 MBq) was injected as a bolus via the lateral tail vein, and dynamic PET imaging (Siemens Inveon preclinical μPET/SPECT/CT system) was performed over 60 min. Immediately afterwards, T1-weighted high-resolution axial, coronary and sagittal brain MRI scans were performed using a Bruker BioSpec 94/30 USR small-animal MR system (Bruker BioSpin GmbH, Karlsruhe, Germany).

## Results

**Fig. 3:**
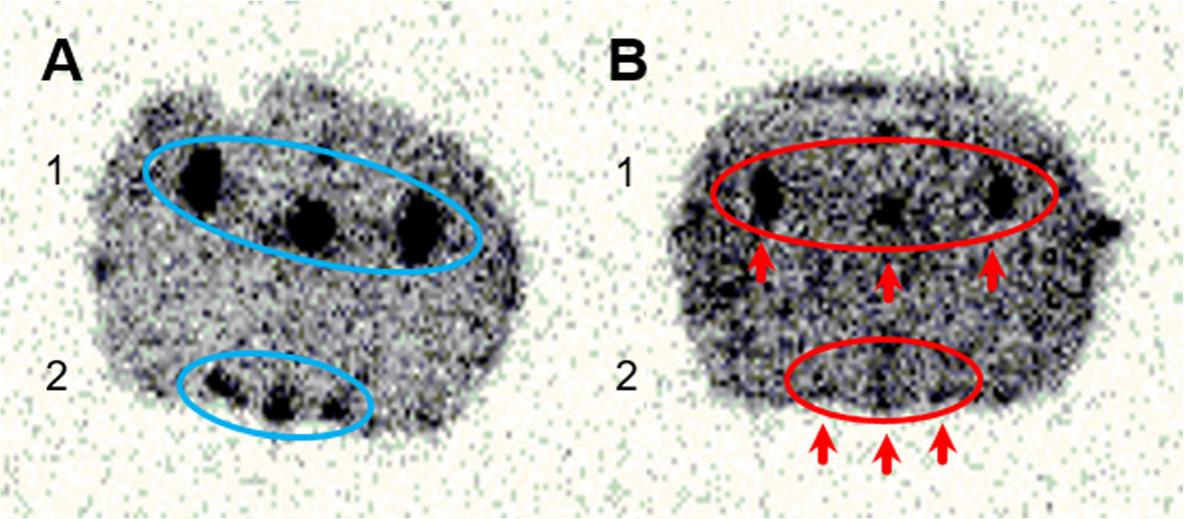
Typical ex vivo brain autoradiography (bregma −2.30 mm) after injection of [^18^F]FE@SNAP into the jugular vein under baseline (**a**) and blocking (**b**) with SNAP-7941 (15 mg/kg). Region *1*: ventricular system; region *2*: hypothalamic area including the third ventricle. *Red arrows* indicate significantly reduced tracer uptake in both regions

Ex vivo autoradiography after IV application of [^18^F]FE@SNAP (baseline group, *n* = 3) showed tracer uptake into the rat brains, with increased accumulation in the lateral hypothalamus and the ventricular system. In the blocking group (*n* = 3), whole-brain uptake was significantly higher than in baseline animals. Specific blocking with 15 mg/kg SNAP-7941 evinced a significant reduction of tracer uptake in the lateral hypothalamus and in the ventricular system (Fig. 3). The ratios are shown in Table 1.

**Table 1:** Calculated ratios of the ROIs (DLU/mm^2^)

Ratio	Baseline	Blocking	*P* values
Hypothalamus/non-target	2.14 ± 0.9	1.58 ± 0.5^a^	0.0004
Ventricle/non-target	2.20 ± 0.4	1.38 ± 0.2^a^	<0.0001

^a^Significant blocking

**Fig. 4:**
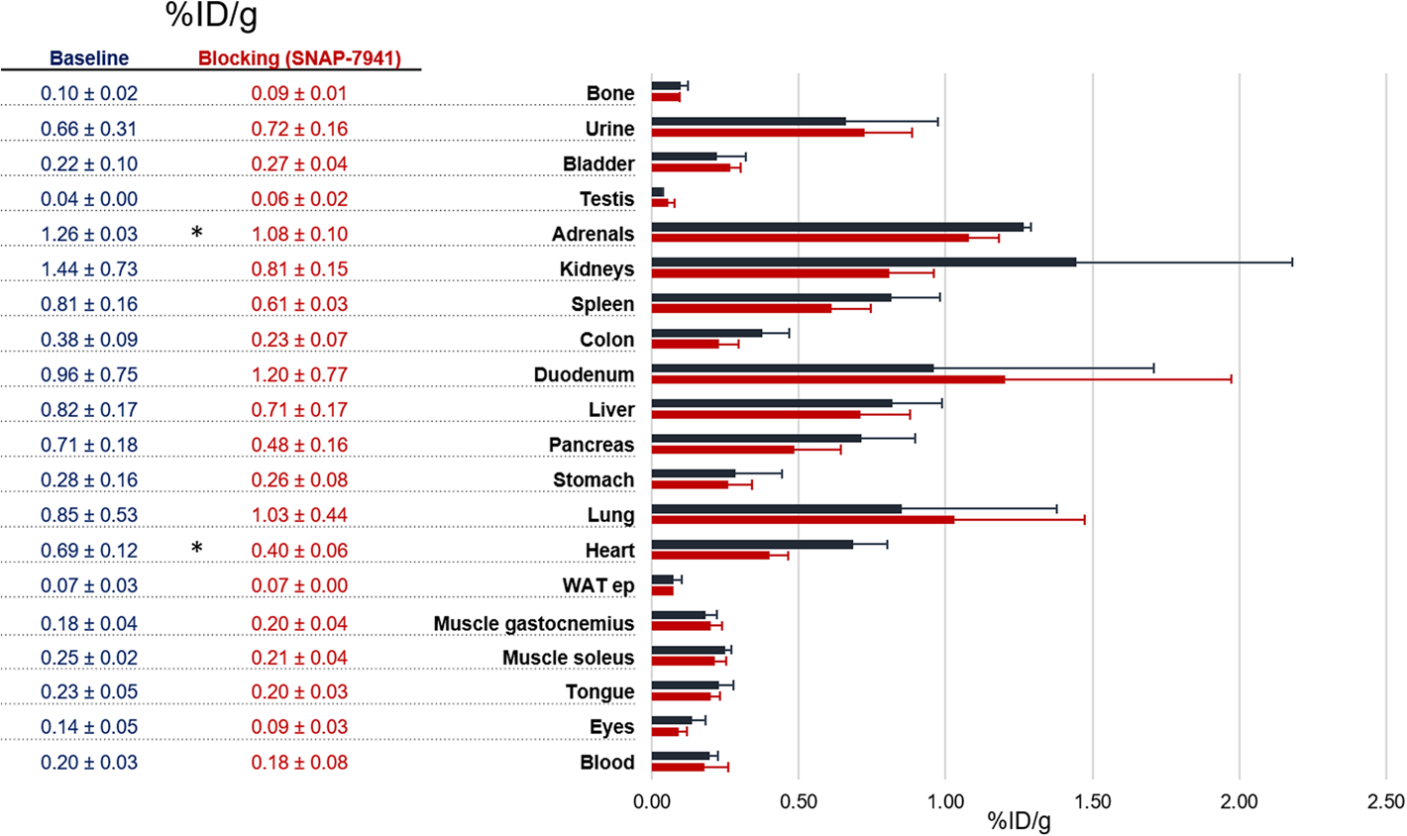
Biodistribution of [^18^F]FE@SNAP. Experiments conducted 45 min after injection in healthy rats under baseline and blocking (15 mg/kg SNAP-7941) conditions. *Bars* represent arithmetic mean ± standard deviation. If not visible, error bars are within the margin of the symbols. *Asterisk* indicates significance

The results of the biodistribution (*n* = 3, each for the “baseline” and the “blocking group”; 45 min after injection) are shown in Fig. 4. Specific blocking with 15 mg/kg SNAP-7941 led to a significant tracer reduction in the heart and adrenals. Other organs known to express MCHR1 to some extent (eye, tongue, muscle soleus, pancreas and colon) showed a trend towards reduced uptake under blocking conditions.

Analysis of metabolites in the blood (*n* = 3) evinced 51.50 ± 5.5 % of the parent compound and 47.73 ± 6.1 % of a hydrophilic radioactive metabolite (probably [^18^F]fluoroethanol) 45 min after tracer application.

**Fig. 5:**
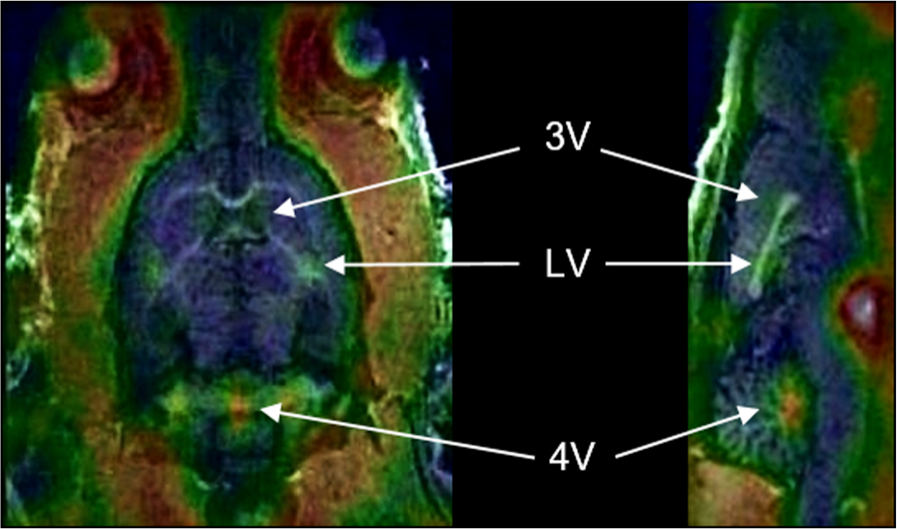
Exemplary small-animal PET/MR images of a rat brain with [^18^F]FE@SNAP. PET data are contributed to summation images from 0 to 90 min. Anatomical structures are indicated by *arrows* (*LV* lateral ventricle, *3V* the third ventricle, *4V* the fourth ventricle). Planes: axial (*left*); sagittal (*right*)

Preliminary small-animal PET measurements in a healthy rat under baseline conditions showed a high tracer uptake in the ventricular system (Fig. 5).

## Discussion

Specific MCHR1 imaging is of high clinical interest for status monitoring in endocrine pathologies like obesity and diabetes. A PET tracer for MCHR1 comprises several advantages for clinicians and patients as the in vivo monitoring and following of the hormone receptor status and related pathologies. Moreover, it could support dose selection of MCHR1 antagonists in drug development [7].

The focus of this study was to investigate the potential of [^18^F]FE@SNAP to specifically label MCHR1-rich regions like the lateral hypothalamus [8]. Therefore, [^18^F]FE@SNAP was injected IV into healthy and conscious rats followed by ex vivo brain autoradiography, biodistribution and analysis of potential blood metabolites.

In order to avoid the well-known effects of anaesthesia [14–16], [^18^F]FE@SNAP was administered directly into the jugular vein of awake and conscious rats. IV application of [^18^F]FE@SNAP showed a high and specific uptake in the ventricular system—where the ependymal cells are recently known to express the MCHR1 [18, 19]—and the hypothalamic region suggesting specific MCHR1 targeting and visualization. Furthermore, blocking of MCHR1 with SNAP-7941 led to a significantly increased overall tracer uptake into the brain, whilst significantly reducing tracer uptake in the presumably MCHR1-rich regions. Since MCHR1-rich regions are blocked with the unlabeled SNAP-7941 and, therefore, specific MCHR1-targeted binding was inhibited, an unspecific tracer uptake in the whole brain was observed.

High specific blocking of the tracer in the brain was not hampered by observed hydrophilic blood metabolites.

Apart from a high specific central MCHR1 uptake, the blocking experiments hinted at a MCHR1-related uptake of [^18^F]FE@SNAP also in the adrenals, eyes, tongue, muscle, pancreas and colon which is in line with literature [3, 20, 21]. However, statistical analysis of the %ID/g of these organs only revealed specific blocking in the adrenals. The specific blocking in the heart raises the question whether MCHR1 might be expressed there too.

With regard to future application of the tracer, it is promising that no defluorination and insignificant uptake of ^18^F-fluoride into the bone were observed.

It is noteworthy that throughout the jugular vein injection, rats were conscious during tracer application and distribution; hence, potential influence of anaesthesia was completely excluded. Therefore, specific binding and blocking resulted directly from the investigated compounds.

The high resolution of ex vivo autoradiography using a Phosphor Imager allowed identification of specific uptake of [^18^F]FE@SNAP in the lateral hypothalamus and in the ependymal cells of the third ventricle epithelium. Hence, as a consequential step, future small-animal PET experiments are feasible.

## Conclusions

Since the MCHR1 is predominantly expressed in the lateral hypothalamus as well as in the ependymal cells of the third ventricle epithelium, a tracer for the MCHR1 should show specific uptake and be significantly blocked by an unlabeled ligand in these areas. [^18^F]FE@SNAP proved these characteristics, which provides strong evidence that it is a highly specific agent for MCHR1 imaging. Involvement of MCHR1 was reported in diabetes and obesity, and MCHR1 has also been related to asthmatic seizures, colitis, depression, anxiety and promotion of sleep. Against this background, [^18^F]FE@SNAP could serve as a useful tool for imaging and therapy monitoring for MCHR1-related pathologies.
